# Stimulating a Mature Body’s Defense System by Maintaining Physical Activity: A Literature Review

**DOI:** 10.1093/geroni/igab046.3260

**Published:** 2021-12-17

**Authors:** Mary Murphy

**Affiliations:** Fairfield University, Fairfield, Connecticut, United States

## Abstract

This review provides summary of research findings on the effects of exercise for changes in the immune system most associated with aging. Immunosenescence is identified as an immune dysregulation with aging that leaves an older adult susceptible to infections and a host of immune-related disorders. Extrinsic modulators of immunosenescence include pathogens, mental stress, nutrition, and exercise. Moderate short acute exercise over time enhances the immune system. Heavy exertion or prolonged exercise bouts may contribute to immunosenescence. In one study, a J-curve result was identified for upper respiratory tract infection. A moderate exercise workload was associated with a 40-50% decrease in upper respiratory tract infections while a 2-6-fold increase was identified among individuals consistently completing heavy exertion. Transient increases of the inflammatory markers of C-reactive protein and Interleukin-6 are noted after excessive exercise. The older adult should consider small increments of change in an exercise load to limit exercise-induced inflammation. These same inflammatory markers are chronically expressed in obese individuals in a resting state. Strategies to manage weight within recommended range to avoid obesity will limit activation of proinflammatory immune cells. In conjunction with physical activity, the lifestyle behaviors that most support immune system health include adequate sleep, nutrition, hydration, and avoidance of excessive alcohol intake. When planning a safe moderate exercise workload, additionally consider hygienic practices to lower transmission of pathogens. Transmission decreases with hand washing, limited hand-to-face contact, distance from large crowds or those with cough, avoiding spaces with poor ventilation and update vaccinations.

